# Extracorporeal membrane oxygenation for avian influenza A (H7N9) patient with acute respiratory distress syndrome: a case report and short literature review

**DOI:** 10.1186/s12890-017-0381-y

**Published:** 2017-02-14

**Authors:** Qi Nie, Ding-yu Zhang, Wen-juan Wu, Chao-lin Huang, Zheng-yi Ni

**Affiliations:** Wuhan Medical Treatment Center, Yintan Road NO.1, Wuhan, 430023 Hu Bei China

**Keywords:** Extracorporeal membrane oxygenation, Avian influenza A H7N9, Acute respiratory distress syndrome

## Abstract

**Background:**

Extracorporeal membrane oxygenation (ECMO) is performed as an acceptable life-saving bridging procedure in patients with severe acute respiratory distress syndrome (ARDS).To patients with avian influenza A (H7N9)-associated ARDS, ECMO could be adopted as a feasible therapeutic solution. We present our successful experience with ECMO utilized in a respiratory failure patient with H7N9 infection.

**Case presentation:**

A 44 years-old female with H7N9-induced ARDS was admitted to intensive care unit (ICU) and was treated with veno-venous ECMO for six days, antiviral therapy, prolonged corticosteroid infusion and other therapies. She suffered significant hemorrhage requiring transfusion of platelets and multidrug-resistant *Acinetobacter Baumannii* infection during ECMO support. Bleeding and infection almost killed the patient's life. Fortunately, she was alive at last and completly recovered after 38 days of ICU stay.

**Conclusions:**

ECMO was effective in this H7N9 patient with a fatal respiratory failure. Mechanical circulatory support was the only chance for our patient with H7N9-associated ARDS to survive until respiratory function recovery. Early detection and rapid response are essential to these serious ECMO-associated complications such as hemorrhage, thrombosis and infection.

## Background

Acute respiratory distress syndrome (ARDS) is a serious hypoxaemia and progressive dyspnea condition which is caused by various direct or indirect factors leading to the development of acute lung injury. Although medical technology is more and more advanced and modern medicine for ARDS has been further systematic in pathological physiology, diagnosis and treatment scheme, mortality rate due to ARDS is still high [1]. The study found that fatality rate of patients with severe ARDS was as high as 62% [2]. Extracorporeal membrane oxygenation (ECMO) is a feasible life-saving support therapy for patients with severe ARDS that is refractory to conventional mechanical ventilation (MV) [3]. Originally applied to support the respiratory function of pediatric patients with good results [4], the use of ECMO has been progressively extended to adult population [5]. Key factors for the indication of ECMO are represented by prognosis of the underlying disease, timing, quality of life of survivors and the possibility of being lung transplant candidate [6]. Main ventilation strategy of ECMO is to allow lungs of patients to rest and maintain theiropening, in order to get opportunity for treatment of lung disease [7].

Occurrence of ARDS in patients with H7N9 infection is associated to an extremely high mortality especially before the introduction of effective antiviral treatment. We present a clinical case of the successful use of ECMO in a patient with H7N9-associated ARDS.

## Case presentation

**Fig. 1:**
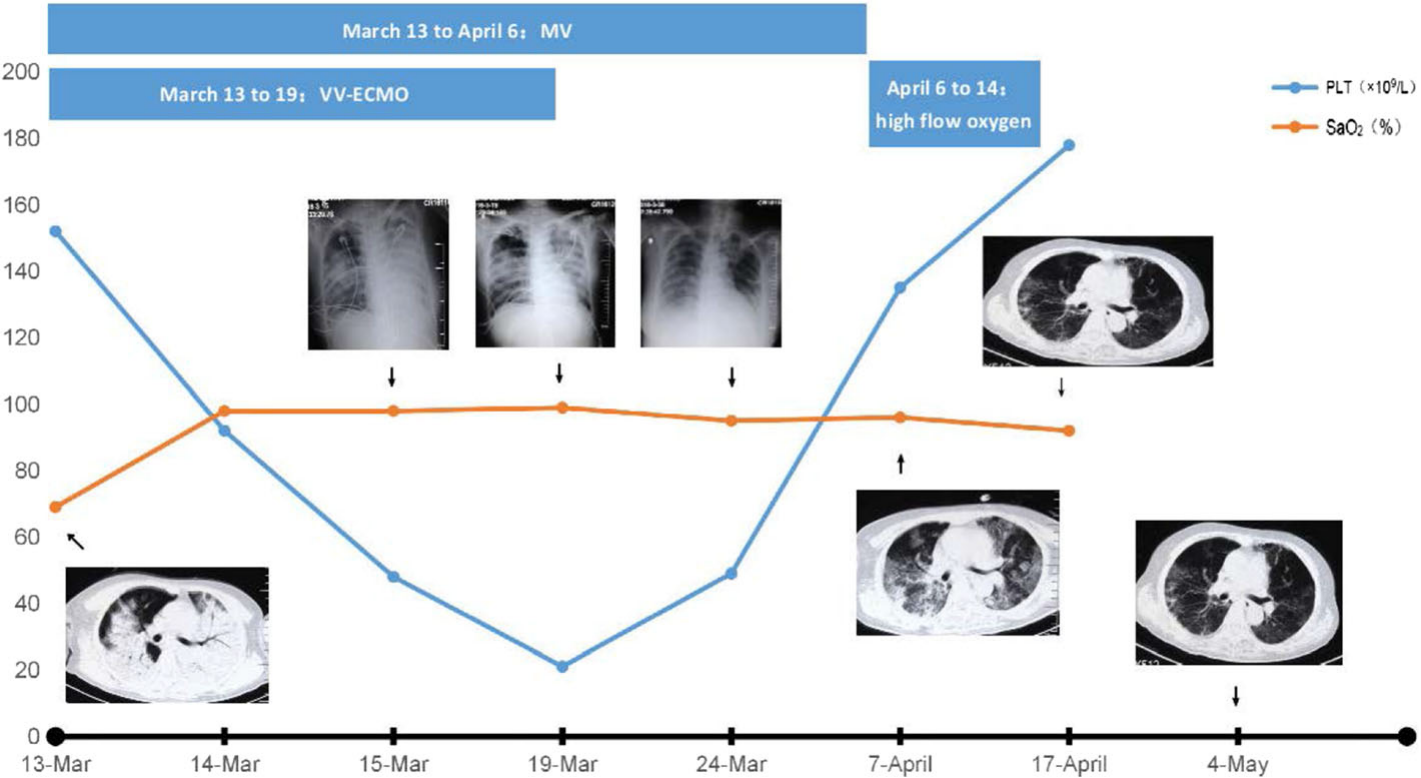
Chest X-Ray(*Bedside*)and computer tomography scans(No PEEP). MV mechanical ventilation,VV-ECMO veno-venous extracorporeal membrane oxygenation. SaO_2_ Arterial Oxygen Saturation(%,NV > 95%)PLT platelet(×10^9^/L)

The patient who had previously been exposed to poultry was a 44 years-old female that had avian Influenza A(H7N9) infection and presented with ARDS. After 15 days of flu-like respiratory symptoms and fever, the patient developed severe respiratory distress and was admitted to the emergency ward of local hospital. Two days after the beginning of treatment with antibiotic, the patient developed mild ARDS.That was initially treated with non-invasive mechanical ventilation (NIV) in the Intensive Care Unit (ICU) of superior hospital. Later, ARDS evolved to the severe stage [8] and a significant septic shock occurred. For this reason, three days after the onset of respiratory failure, the patient received endotracheal intubation and mechanical ventilation(MV).The patient was referred to ECMO center of our hospital in March 13, 2016 since conventional protective MV did not improve the patient's condition..Severe ARDS (PaO2/FiO2 = 46 mmHg) due to pneumonia caused by avian influenza A(H7N9) virus was diagnosed since the bronchial lavage fluid (BLF) and pharynx swab were positive for H7N9-RNA. Chest X-ray and CT scan showed diffuse and bilateral pulmonary real change opacities (Fig. 1).

Upon arrival at the referral ECMO center, sepsis, septic shock, acidosis and so forth, were treated and veno-venous ECMO (VV-ECMO) was initiated (18 F cannula was inserted into right jugular vein and right femoral vein, respectively). The by-pass was established with a blood flow of 4.13 L/min and sweep gas of 4 L/min. This strategy offered the advantage to rest the lung and minimize the risk of ventilator-induced lung injury [9]. Details of antiviral therapy, steroid therapy, antibiotic,antifungal treatments and so on are shown in Table 1.

**Table 1:** Main clinical characteristics of patient

Clinical characteristics	
Diagnosis at admission	avian influenza A(H7N9),ARDS,Sepsis,Septic shock
H7N9-RNA (BLF&pharynx swab)	From March 13th to March 29th:positive
*Acinetobacter Baumannii*	From March 17 to March 25:positive
Respiratory support (outer court)	Time from symptoms onset to NIV (15 days);
	Time from NIV to MV initiation (1 day);
	Time from MV initiation to VV-ECMO initiation (2 days)
Respiratory support (our hospital)	March 13 to 19:VV-ECMO duration
	March 13 to April 6:MV
	April 6 to 9:high flow oxygen through trachea cannula,oxygen flow rate:50 ~ 55 L/min
	April 9 to 14:high flow oxygen through face mask,oxygen flow rate:35 ~ 40 L/min
	April 14 to 17:administering oxygen inhalation through nasal catheter,oxygen flow rate:2-6 L/min
	April 17 to 20:stop oxygen inhalation
Antiviral therapy	March 13 to April 1:Oseltamivir 150 mg,Bid
Anti infection treatments	March 13 to April 16:Tienam 1.0 g,Tid;Tigecycline 50 mg,Bid;Moxifloxacin 400 mg,Qd.etc. Adjust medication according to the results of culture and drug sensitivity.
Antifungal therapy	March 13 to April 11:Micafungin 50 mg,Qd.
steroid therapy	March 13 to April 11:methylprednisolon 80 mg,Bid,gradually reduced dosage.

With the application of ECMO, the patient's blood gas analysis results gradually improved (Table 2). Chest radiography was taken every day. In the first radiograph (Fig. 1), opacities and consolidation of almost the entire left lung were demonstrated as “white lung”. However, the opacities and consolidation disappeared gradually (Fig. 1) when the infection was controlled, and the number of white cells returned to normal too.

**Table 2:** Ventilator and ECMO parameters

Variable	March 13(no ECMO) at admission	March 14 ECMO(2nd day)	March 19 ECMO((7th day)	March 21 stop ECMO(3rd day)
vital sign	T:36.7,HR:129, BP:74/36	T:35.8,HR:76, BP:119/81	T:36.5,HR:77, BP:122/79	T:36.9,HR:68, BP:132/67
MV mode	SIMV	PCV-SIMV	SIMV	SIMV
PEEP	18	8	12	18
FiO_2_	100%	40%	60%	70%
f	20	10	12	16
SaO_2_	69% ~ 83%	98%	99%	97%
Blood Flow	--	3.46 L/min	3.00 L/min	--
Sweep Flow	--	2.50 L/min	3.00 L/min	--
PH	7.274	7.315	7.440	7.468
PaO_2_	6.92	13.41	18.00	27.20
PaCO_2_	5.69	4.92	6.06	6.50

T temperature(°C),HR heart rate(bpm),BP blood pressure(mmHg),MV mechanical ventilation,SIMV synchronized intermittent mandatory ventilation,PCV Pressure Control Ventilation,PEEP Positive End Expiratory Pressure (cmH_2_O),FiO_2_ Fraction of inspired oxygen(%),F mechanical ventilation frequency(bpm),SaO_2_ Arterial Oxygen Saturation(%,NV > 95%),PH value(7.35-7.45),PaO_2_ Partial pressure of oxygen in arterial blood (kPa,NV:10.66-13.33),PaCO_2_ Partial pressure of carbon dioxide in arterial blood (kPa,NV:4.65-5.98)

Treatment with ECMO was maintained for 6 days and stopped due to severe thrombocytopenia because heparin must be applied when starting ECMO. Subcutaneous hemorrhage occurred in fingers and left thigh of the patient. Bloody liquid was sucked out from bronchi. Platelet antibodies were detected in blood and platelet transfusion was ineffective. Fortunately, platelets gradually returned to normal after the withdrawal of ECMO (Fig. 1). At the same time, multidrug-resistant *Acinetobacter Baumannii* infection was found in BLF, sputum and blood. It was fortunate that infection and thrombocytopenia were found in time and gradually cured. On April 9, the patient was successfully extubated and ventilated with non-invasive ventilation (NIV) by face-mask. After 11 days, the patient was discharged to a step down ICU and then at home. After more than eight months of follow-up, the pharynx swab H7N9-RNA remained negative and she was alive finally with a good physical condition.

## Discussion

Favorable outcomes in severe ARDS have been reported with ECMO use during the 2009 H1N1 influenza pandemic, which led to increased interest and use of ECMO for refractory respiratory failure. VV-ECMO can supply sufficient pulmonary support when gas exchange is severely compromised and presents an ultimate option in problematic cases. However, indications for VV-ECMO are not unequivocal and it is highly invasive, presenting several specific problems and intellectual concepts that require understanding in caring for these patients. Since VV-ECMO is highly invasive and associated with numerous potential complications, such as hemorrhage, thrombosis and infection, its use should only be considered in patients with a high probability of death with conventional treatment.

In fact, H7N9-associated ARDS carries a high mortality rate. However, the care of patients with H7N9 infection has advanced since the introduction of ECMO, with increased life quality and decreased mortality. We performed a retrospective observational analysis of our experience with ECMO support for H7N9-associated ARDS. In this report, ECMO is a crucial extracorporeal life support in the H7N9 infection patient with severe ARDS.

The features of severe H7N9 avian flu patients are viral infections, dyspnea, respiratory failure and ARDS, it final developed to multiple organ failure and life-threatening. Therefore, in addition to antiviral therapy(oseltamivir), anti- multiple bacterial infections (application of antibiotics and anti-fungal drugs), glucocorticoid (methylprednisolone) and the symptomatic treatment, the timely and effective respiratory support is particularly important in treatment of severe avian flu patients.

Our patient has entered a stage of ARDS when she was hospitalized. Since mechanical ventilation by tracheal intubation could not improve patient with hypoxia, soon organ failure must occur in brain, heart, liver, kidneys and other vital organs of high oxygen demand. Emergency VV-ECMO partial instead of lung ventilation and oxygenation rapidly improved oxygen supplement of the body organs. It avoided the occurrence of multiple organ failure, decreased support of respiratory machine, reduced ventilator-associated lung injury, for lungs to get adequate rest, thus won time for treatment of the primary disease.

Evidence supporting ECMO as life saving treatment for adults with respiratory failure are growing [10]. However, ECMO is a costly intervention that may carry the risk of futility and serious side effects such as major bleeding and infection. During ECMO support, as heparin must be applied at the same time, so thrombocytopenia and coagulopathy inevitably arise in patients. ECMO was started at 13:20 on March 13 for our patient, dramatic decrease in platelets (PLT 48x10^9^/L) was found on March 15. Skin ecchymosis was appeared in the groin where catheters were placed on March 19, subcutaneous hemorrhage occurred in fingers and left thigh of the patient, platelet had fallen to 21 × 10^9^/L. But more worrying was that bloody liquid was sucked out from bronchi and platelet transfusion did not work. Through discussion with experts, ECMO was withdrawn at 17:05 on March 19, and thrombopoietic treatment and platelet monitoring were continued, finally, the platelet returned to the normal range on April 3. Infection is another side effect of the application of ECMO. Four days after hospitalization, BLF, sputum and blood stream infection with multidrug-resistant *Acinetobacter Baumannii* occurred in our patient, which could lead to septic shock and death. Fortunately, we discovered and prevented the progress of infection in time.

Under these circumstances, indication and selection criteria need to be better defined.In our report, ECMO was used in severe, life threatening respiratory failure caused by a curable bird flu disease. Extracorporeal Life Support Organization (ELSO) guide-lines suggest ECMO when the risk for mortality is at least 50% (as identified by PaO2/FiO2 < 150 and or Murray score of 2–3) [11]. In this case, VV-ECMO was performed because PaO2/FiO2 ratio of the patient was only 46 mmHg. Fact proved that VV-ECMO for H7N9-associated respiratory failure gave a good result.

## Conclusions

ECMO was a feasible salvage modality in this H7N9 patient with an fatal respiratory failure.ECMO is not the only factor to improve the condition of patient, the combined effect of hormone and antiviral therapy, anti-shock therapy helps patient to survive. It was also important to discover in time and rapid response serious ECMO-associated complications. Our patient was cured and went back to her previous quality of life at last, thanks to ECMO giving her time to get treatment. This case suggests that a multidisciplinary team, involving critical care and infectious diseases experts, is very critical to define whether ECMO is an adequate therapeutic option also in patients with severe pulmonary infectious diseases. We recommend treatment with ECMO as early as possible, but more clinical evidence is required to support this view. Further studies are needed to understand how to best utilize and optimize ECMO therapy in patients with ARDS. In short, our successful treatment of patients with severe H7N9 avian flu should be credited to application of ECMO and important ventilation adjustment according to patient's condition. Retrospective analysis of comprehensive treatment of this patient provided a reliable reference to treatment of ARDS and severe pneumonia.

## Abbreviations

ARDS: Acute respiratory distress syndrome
BLF: Bronchial lavage fluid
CT: Computed tomography
ECMO: Extracorporeal membrane oxygenation
ICU: Intensive care unit
MV: Mechanical ventilation
NIV: Non-invasive mechanical ventilation
VV-ECMO: veno-venous ECMO
